# Sequential Growth of 2D/3D Double‐Layer Perovskite Films with Superior X‐Ray Detection Performance

**DOI:** 10.1002/advs.202102730

**Published:** 2021-09-08

**Authors:** Xiuwen Xu, Wei Qian, Jian Wang, Jiecheng Yang, Jianwei Chen, Shuang Xiao, Yongshuai Ge, Shihe Yang

**Affiliations:** ^1^ Guangdong Key Lab of Nano‐Micro Material Research School of Chemical Biology and Biotechnology Shenzhen Graduate School Peking University Shenzhen 518055 China; ^2^ Institute of Biomedical Engineering Shenzhen Bay Laboratory Shenzhen 518055 China; ^3^ Research Center for Medical Artificial Intelligence Shenzhen Institute of Advanced Technology Chinese Academy of Sciences Shenzhen 518055 China; ^4^ Paul C. Lauterbur Research Center for Biomedical Imaging Shenzhen Institute of Advanced Technology Chinese Academy of Sciences Shenzhen 518055 China

**Keywords:** direct X‐ray detection, interfacial engineering, low‐dimensional perovskites

## Abstract

Perovskite materials in different dimensions show great potential in direct X‐ray detection, but each with limitations stemming from its own intrinsic properties. Particularly, the sensitivity of two‐dimensional (2D) perovskites is limited by poor carrier transport while ion migration in three‐dimensional (3D) perovskites causes the baseline drifting problem. To circumvent these limitations, herein a double‐layer perovskite film is developed with properly aligned energy level, where 2D (PEA)_2_MA_3_Pb_4_I_13_ (PEA=2‐phenylethylammonium, MA=methylammonium) is cascaded with vertically crystallized 3D MAPbI_3_. In this new design paradigm, the 3D layer ensures fast carrier transport while the 2D layer mitigates ion migration, thus offering a high sensitivity and a greatly stabilized baseline. Besides, the 2D layer increases the film resistivity and enlarges the energy barrier for hole injection without compromising carrier extraction. Consequently, the double‐layer perovskite detector delivers a high sensitivity (1.95 × 10^4^ *μ*C Gy_air_^−1^ cm^−2^) and a low detection limit (480 nGy_air_ s^−1^). Also demonstrated is the X‐ray imaging capacity using a circuit board as the object. This work opens up a new avenue for enhancing X‐ray detection performance via cascade assembly of various perovskites with complementary properties.

## Graphical abstract

By integrating a layered 2D perovskite with a vertically crystallized MAPbI_3_, a double‐layer perovskite is constructed for direct X‐ray detection, showing stable baseline, a high sensitivity of 19 503 *μ*C Gy_air_^−1^ cm^−2^, and a low detection limit of 480 nGy_air_ s^−1^. This work provides a strategy to unlock the performance limitations stemming from the intrinsic properties of the perovskite.

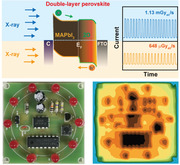

## 1 Introduction

X‐ray detection has become increasingly important for widespread applications, including medical imaging, non‐destructive industrial inspection and security screening.^[^ ^1^ ^]^ According to the working mechanism, X‐ray detectors are divided into indirect and direct modes. Compared with the indirect mode wherein X‐ray is first converted to light by a scintillator, which is then detected by a photodiode, the direct one is capable of directly converting X‐ray to electronic signals, thus offering higher spatial resolution and simpler device configuration.^[^ ^2^ ^]^ Thanks to the ease of large‐area fabrication on the thin‐film transistor (TFT) based electronic readout, amorphous selenium (ɑ‐Se) has dominated the market of direct‐conversion X‐ray detectors ever since the dawn of this century. Nonetheless, due to the low X‐ray attenuation coefficient of ɑ‐Se, such detectors are limited to the application scenarios of soft X‐ray, such as mammography. In addition, ɑ‐Se suffers from poor carrier transport, which leads to a low sensitivity of 20 µC Gy_air_^−1^ cm^−2^, along with a high limit of detection (LoD) of 5500 nGy_air_ s^−1^.^[^ ^3^ ^]^ Since sensitivity and LoD are the most important figures‐of‐merit for X‐ray detectors, it is imperative to develop new materials with a higher sensitivity and a lower LoD for the next‐generation X‐ray detectors, which will enable sharp image acquisition at an ultralow‐dose of X‐ray and thus minimize the risk of radiation exposure.^[^ ^4^ ^]^

Recently, halide perovskite materials are emerging as a dark horse in direct X‐ray detection for their intriguing optoelectronic properties, including high X‐ray attenuation coefficient and large carrier mobility‐lifetime (*µτ*) product, on top of their easy solution processability.^[^ ^3^, ^5^ ^]^ With less than 5 years’ development, tremendous progress has been made in exploring different perovskite materials for direct X‐ray detection. Among these materials, 3D perovskites, such as MAPbI_3_,^[^ ^4^, ^6^ ^]^ MAPbBr_3_,^[^ ^7^ ^]^ CsPbBr_3_,^[^ ^8^ ^]^ etc., are advantageous in achieving high sensitivity due to their large *µτ* products. For example, a record sensitivity of 1.22 × 10^5^ and 2.4 × 10^4^ *μ*C Gy_air_^−1^ cm^−2^ has been achieved with MAPbI_3_ and MAPbBr_3_, respectively.^[^ ^6^, ^7^ ^]^ However, the high sensitivity of 3D perovskites is overshadowed by the severe baseline drifting problem arising from ion migration in the material, which will prevent accurate signal recording for practical use.^[^ ^3^, ^6^, ^7^, ^8^, ^9^ ^]^ Very recently, it has come to our notice that the notorious baseline drifting problem can be well addressed by using low‐dimensional perovskites. In particular, due to the intrinsically suppressed ion migration, a 2D BDAPbI_4_ (BDA = NH_3_C_4_H_8_NH_3_) perovskite and a 0D MA_3_Bi_2_I_9_ perovskite both showed a rather stable baseline even when subjected to continuous operation for hours.^[^ ^10^ ^]^ Moreover, the BDAPbI_4_ and MA_3_Bi_2_I_9_ presented a low LoD of 430 and 83 nGy_air_^−1^ s^−1^, respectively. As defined by the International Union of Pure and Applied Chemistry (IUPAC), the LoD is the minimum dose rate for a detector to yield a signal‐to‐noise ratio (SNR) of 3. Given the moderate sensitivity of these detectors (BDAPbI_4_: 242 *μ*C Gy_air_^−1^ cm^−2^; MA_3_Bi_2_I_9_: 1947 *μ*C Gy_air_^−1^ cm^−2^), the low LoD is attributed to small dark current, which stems from the large film resistivity.^[^ ^10^ ^]^ Since shot noise is directly determined by dark current, reducing dark current, and thus the shot noise level, will increase the SNR.^[^ ^11^ ^]^ Unfortunately, despite the great success of the low‐dimensional perovskites in stabilizing the baseline and in reducing the LoD, their sensitivity is largely limited by the intrinsically poor carrier transport properties and thus thrown into the shade by their 3D counterparts.^[^ ^11^, ^12^ ^]^

Naturally, we reason that a judicious combination of the 2D and 3D perovskites could get the best of the two worlds and thus has the potential to unlock the limitations in X‐ray detection imposed by the intrinsic properties of the individual perovskite alone. Of note, both 2D/3D mixed perovskites and 2D‐capped 3D perovskites with a thickness of ≈500 nm, typically prepared by spin coating, have been extensively developed for solar cells and photodetectors.^[^ ^13^ ^]^ However, perovskite films for X‐ray detectors have to have a thickness of at least tens of micrometers to ensure sufficient X‐ray absorption, thus ruling out most of the existing perovskite solution processing methods, including spin coating.^[^ ^14^ ^]^ As far as we know, neither 2D/3D mixed perovskites nor 2D‐capped 3D perovskites have been reported for direct X‐ray detection, not mentioning the vertically assembled 2D/3D double‐layer perovskites we propose here. It has been a challenge to prepare thick 2D/3D mixed perovskite films with an ordered and gradient phase distribution to ensure directional carrier transport by virtue of the favorably aligned energy levels in the bulk. Meanwhile, for the 2D‐capped 3D perovskite films, the 2D perovskite capping layer is commonly made of large‐sized ammonium salts or amines beneath or atop the 3D perovskite, and this layer is generally thin with an uncontrollable thickness.^[^ ^15^ ^]^ Although the ultrathin 2D perovskite capping layer can tune the surface defect states and interfacial energy level alignment for some optoelectronic devices, it is questionable if it is sufficiently robust for X‐ray detectors here that need to sustain high electrical fields and X‐ray radiation.

Distinct from the 2D/3D mixed and 2D‐capped 3D perovskites, the double‐layer perovskite is proposed to be constructed by stacking the 2D and then the 3D perovskites in sequence, and this allows a better control of the 2D and 3D perovskite layers (e.g., thickness, phase distribution, etc.) individually and separately without interfering with each other.^[^ ^16^ ^]^ A relevant work was recently reported by Jang et al. on the construction of an intact 2D/3D double‐layer perovskite by a solid‐state in‐plane growth method, where 2D perovskite (*n* = 1) was placed on 3D perovskite and hot‐pressed at 60 MPa. Benefiting from the properly tailored local electric field at the 2D/3D junction, the double‐layer perovskite solar cell offers a high efficiency of 24.59%.^[^ ^16b^ ^]^ Another work along the line was by Amratisha et al. using a spray coating protocol with two airbrushes. They sequentially sprayed 3D perovskite and quasi‐2D perovskite (*n* = 50) to get the double‐layer perovskite. However, due to the lack of process control, the as‐fabricated double‐layer perovskite solar cell merely delivered an efficiency of 2.7%, only slightly higher than the 3D counterpart (2.3%), indicative of the poor film quality.^[^ ^16a^ ^]^ Very recently, we developed an aerosol–liquid–solid (ALS) process and performed in‐depth investigations of the perovskite formation mechanisms, which enabled the fabrication of high‐quality, thick perovskites films essential for X‐ray detectors. Importantly, the ALS technology has been advanced to such extent that sequential growth of perovskites with different compositions becomes feasible, laying a foundation for the present work in controllably assembling 2D/3D double‐layer perovskite films for direct X‐ray detection.^[^ ^17^ ^]^

In this article, we adopt the ALS process to develop an innovative direct‐conversion X‐ray detector based on a 2D/3D double‐layer perovskite film, where a 2D (PEA)_2_MA_3_Pb_4_I_13_ layer parallel to the substrate is cascaded with a vertically grown 3D MAPbI_3_ layer. It was found that the 2D perovskite remarkably mitigates the notorious baseline drifting problem by suppressing ion migration in the double‐layer perovskite films. Moreover, the 2D layer inhibits hole injection from the electrode while guaranteeing efficient carrier extraction, and at the same time increases the film resistivity. Due to properly aligned interfacial energy level and a judicious combination of the 2D and 3D perovskites with complementary properties, the 2D/3D double‐layer perovskite stands out from the 3D perovskite, 2D/3D mixed perovskite and 2D/3D/2D sandwich perovskite, offering the state‐of‐the‐art X‐ray detection performance, with a high sensitivity of 1.95 × 10^4^ *μ*C Gy_air_^−1^ cm^−2^ and a low LoD of 480 nGy_air_ s^−1^. In addition, the X‐ray imaging of a printed circuit board is demonstrated. This work opens a new way to unlock the intrinsic limitations of the perovskites, and will fuel the future development of perovskite X‐ray detectors.

## 2 Results and Discussion

### 2.1 Formation of 2D/3D Double‐Layer Perovskite Films

**Figure 1:**
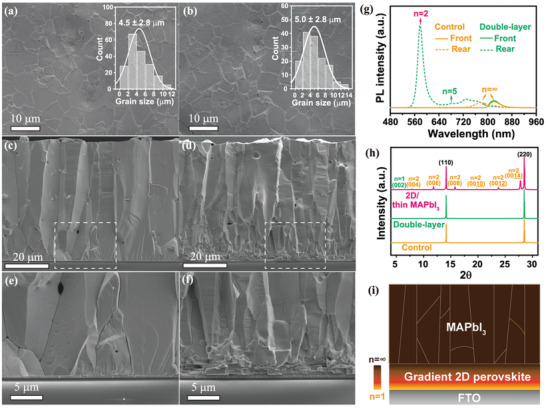
a,c,e) SEM images of the control and b,d,f) double‐layer perovskite films, the inset in (a) and (b) shows the distribution of the grain sizes; g) Steady‐state PL spectra of the control and double‐layer perovskite films; h) The XRD patterns of the control, double‐layer perovskite and 2D/thin MAPbI_3_, where the double‐layer perovskite is 1.6 *μ*m thick (PEA)_2_MA_3_Pb_4_I_13_ with 60 *μ*m thick MAPbI_3_ atop while the 2D/thin MAPbI_3_ denotes 1.6 *μ*m thick (PEA)_2_MA_3_Pb_4_I_13_ with 2 *μ*m thick MAPbI_3_ atop; i) Schematic diagram illustrating the structure of double‐layer perovskite films, where the gradient 2D perovskite is prepared by a precursor solution with a nominal composition of (PEA)_2_MA_3_Pb_4_I_13_.

The 2D/3D double‐layer perovskite film was prepared by successively depositing a 2D perovskite with a nominal composition of (PEA)_2_MA_3_Pb_4_I_13_ (thereafter denoted as (PEA)_2_MA_3_Pb_4_I_13_) and 3D perovskite MAPbI_3_ on a fluorine‐doped SnO_2_ (FTO) glass using the ALS method,^[^ ^17^, ^18^ ^]^ and a full 3D film of MAPbI_3_ with a similar thickness was also prepared as the control sample. As shown in top‐view SEM images (**Figure** **1**a,b), the control and double‐layer perovskite films show a quite similar surface morphology with an average grain size of 4.5 and 5 *μ*m, respectively. However, the cross‐sectional morphology of the double‐layer perovskite differs substantially from that of the control. It is found that in the control sample, the 60 *μ*m thick MAPbI_3_ tend to preferentially and continuously crystallized along the direction perpendicular to the substrate (Figure 1c,e). Such a preferred vertical growth of the thick MAPbI_3_ film stems from its crystallization mechanism in the ALS process, which has been revealed in our recent work.^[^ ^17^ ^]^ Briefly, in the ALS process, the perovskite crystallization process is streamlined through the three adjoined layers of aerosol/liquid/solid phases in a steady state. By intentionally avoiding the perovskite nucleation from the top (aerosol/liquid) interface, the perovskite can grow smoothly at the bottom (solid–liquid) interface sustained by the continuous solute supply from the top (aerosol/liquid interface). This unique process facilitates the vertical growth of perovskite films to any thickness, at least in theory. By contrast, in the horizontal direction, the perovskite growth is more random and the vertical growth induced strain inevitably leads to the grain formation and coarsening. Thus, the continuous crystal growth along the vertical direction versus the grain boundary formation to accommodate strain along the horizontal direction results in the preferred vertical growth of the 3D perovskite.^[^ ^17^ ^]^ Strikingly, in the double‐layer perovskite, a well‐defined layer‐structured perovskite (thickness: 1.6 *μ*m) oriented parallel to the substrate is clearly observed underneath the vertically grown MAPbI_3_ (Figure 1d,f), which is attributed to the 2D (PEA)_2_MA_3_Pb_4_I_13_. Notably, such a layered structure is rather different from and unseen in the 2D perovskite films prepared by the one‐shot spin‐coating method,^[^ ^19^ ^]^ which is expected to be beneficial for the suppression of ion migration.^[^ ^20^ ^]^

Steady state photoluminescence (PL) measurements were conducted to investigate the control and double‐layer perovskites (Figure 1g). As expected, when the perovskites are excited from the front (perovskite) side, the control and double‐layer perovskite both show a PL emission peak at 820 nm, which is attributed to the MAPbI_3_. The obviously red‐shifted PL as compared to that reported for MAPbI_3_ thin films (780 nm) is ascribed to the relatively rough surface of the thick perovskite (Figure S1, Supporting Information), which induces significant photon reabsorption effect given the small critical angle (23.5°) at the perovskite/air interfaces.^[^ ^21^ ^]^ When the perovskites are excited from the rear (FTO) side, the control shows a typical PL emission peak of MAPbI_3_ at 780 nm. In contrast, the double‐layer perovskite exhibits multiple PL emission peaks of low‐dimensional perovskite phases of PEA_2_(MA)*_n_*_−1_Pb*_n_* I_3_ *_n_*_+1_, such as *n* = 2 (577 nm) and *n* = 5 (680 nm), consistent with the previous reports.^[^ ^22^ ^]^ Furthermore, X‐ray diffraction patterns (XRD) were acquired to study the crystal structure of the perovskites (Figure 1h). It is interesting to note that the control and double‐layer perovskites show almost identical XRD patterns, with two predominated peaks at 14.1° and 28.2°, indicating that the MAPbI_3_ preferentially grows along the [110] direction perpendicular to the substrate. Notably, the MAPbI_3_ film with such a preferred vertical crystallization direction outperforms that with a random crystallographic orientation in terms of enabling efficient charge transport and extraction because of the reduced grain boundaries and intra‐grain defects along this direction.^[^ ^23^ ^]^ The absence of the peaks of low‐dimensional perovskite phases in double‐layer perovskite is caused by the thick MAPbI_3_ (60 *μ*m) layer that prevents the X‐ray from penetrating into the underlying (PEA)_2_MA_3_Pb_4_I_13_. This is confirmed by the XRD patterns of the (PEA)_2_MA_3_Pb_4_I_13_ with a thin MAPbI_3_ layer (2 *μ*m) atop, which exhibit diffraction peaks corresponding to the (00l) plane of the low‐dimensional perovskite phases.^[^ ^24^ ^]^ These results prove the existence of various low‐dimensional perovskite phases in the bottom layer of the double‐layer perovskite films.

To gain a deeper insight into the bottom layer, we took a closer look at the layered 2D (PEA)_2_MA_3_Pb_4_I_13_ itself with regard to the formation mechanism and structural properties. Given the pivotal effects of the coordination complexes in the precursor solution on perovskite crystallization,^[^ ^25^ ^]^ we first characterized the precursor solution of (PEA)_2_MA_3_Pb_4_I_13_ and MAPbI_3_ to check whether the PEA cation induces the formation of different coordination complexes in the solution (Figure S2a,b, Supporting Information). It turns out that the absorption and PL emission spectra of the two solutions are almost identical, indicative of rather similar coordination complexes (e.g., [PbI_4_]^2−^, [PbI_3_]^–^, etc.) in the solutions.^[^ ^26^ ^]^ Thus, we reason that the crystallization of the (PEA)_2_MA_3_Pb_4_I_13_ is ruled by the competition between MA and PEA in reacting with these coordination complexes. Then the crystallization kinetics of a sprayed wet (PEA)_2_MA_3_Pb_4_I_13_ film (heated at 140 °C) was monitored by in‐situ PL spectroscopy. As shown in Figure S2c,d (Supporting Information), the PL emission peak of 3D perovskite first shows up at 20 s and reaches its maximum intensity at 40 s. Coincidently, the PL emission peaks of *n* = 1 and *n* = 2 phases start to occur at 40 s and increase with prolonged annealing time. This phenomenon strongly proves that in the wet (PEA)_2_MA_3_Pb_4_I_13_ film, 3D perovskite first crystallizes, consuming most of MA. Subsequently, PEA participates in reacting with the coordination complexes, mainly forming the *n* = 1 and *n* = 2 phases due to the limited MA remaining.

In addition, SEM images of the (PEA)_2_MA_3_Pb_4_I_13_ films (nominal composition) prepared with different cycles of the ALS process were acquired. It is found that with 2 cycles of ALS process, the full film coverage has not been achieved, and instead, we see an intriguing morphology with cubic crystals atop thin crystal layers (Figure S3a, Supporting Information). By using an energy‐dispersive X‐ray spectroscopy (EDS), the atomic ratio of I to Pb of the cubic crystal and the thin crystal layer is determined to be 3 and 3.8, respectively (Figure S4, Supporting Information). Thus, the cubic crystal is attributed to the 3D (MAPbI_3_) phase, whereas the thin crystal layer is *n* = 1 ((PEA)_2_PbI_4_) or *n* = 2 ((PEA)_2_MAPb_2_I_7_) or their mixed phases. This provides strong evidence that the 3D perovskite is first crystallized at the liquid/air interface due to solvent evaporation at the interface, lending the (PEA)_2_MA_3_Pb_4_I_13_ film a gradient phase distribution, with 3D perovskite atop and the low‐*n*‐number phases located at the bottom. It is understandable and important to notice that these low‐*n*‐number phases with the characteristic 2D structure lie down laminating on the substrate, and render the (PEA)_2_MA_3_Pb_4_I_13_ to form a clearly visible layered structure (Figure S3, Supporting Information). Moreover, with additional cycles of the ALS process, the (PEA)_2_MA_3_Pb_4_I_13_ film continues to grow mainly following the dissolution‐recrystallization mechanism until the complete film coverage at a thickness of 1.6 *μ*m.^[^ ^17^, ^27^ ^]^ Meanwhile, grains of the 3D phase are gradually transformed from rectangle to irregular polygon as a result of the minimization of surface energy.

For a more direct demonstration of the 2D/3D transition, we performed cross‐sectional SEM coupled with EDS at the interface (Figure S5, Supporting Information). As expected, the I/Pb atomic ratio at the bottom is 4.2, and it gradually decreases to 3.8, 3.5, and about 3 from the bottom to the top, again conforming to the gradual change of the dominating phase from (PEA)_2_PbI_4_ (*n* = 1) to (PEA)_2_MAPb_2_I_7_ (*n* = 2) and to the mixed‐dimensional perovskite (high *n*) close to MAPbI_3_. We attribute those regions with I/Pb atomic ratio of about 3 to the mixed‐dimensional perovskite, since with a continuous supply of precursor solution containing PEAI in the ALS process, the participation of PEAI in the perovskite crystallization cannot be completely excluded. A rough estimation of their vertical distribution is indicated in Figure S5g (Supporting Information), with about 180 nm for *n* = 1, 510 nm for the mixture of *n* = 1 and *n* = 2, and 1000 nm for the mixed 2D/3D perovskite (high *n*). Though we do not see a clear 2D/3D boundary, the vertical phase distribution featured by the *n*‐gradient has been clearly validated. Since the layer structured (PEA)_2_MA_3_Pb_4_I_13_ is introduced in the hope to suppress ionic migration while optimizing the energy level alignment in the device, the 1.6 *μ*m thick (PEA)_2_MA_3_Pb_4_I_13_ interlayer with full film coverage was then taken for the growth of the double‐layer perovskite to achieve such goals.

The crystal orientation of the 1.6 *μ*m thick (PEA)_2_MA_3_Pb_4_I_13_ is evaluated by XRD measurement. As indicated in Figure S6a (Supporting Information), (PEA)_2_MA_3_Pb_4_I_13_ exhibits a set of diffraction peaks at low angles (<14°), corresponding to the (00l) crystal plane of *n* = 1 and *n* = 2 phases, indicating the existence of the parallel orientation of these 2D perovskite phases.^[^ ^19^, ^28^ ^]^ Thus, the charge transport will be compromised to some extent by the insulating PEA spacer layer, but the increased resistivity as a barrier layer is expected to reduce detector noise. Particularly notable is the absence of the characteristic peaks of intermediate *n*‐number (3 < *n* < ∞) phases at low diffraction angles (2*θ* < 14°). This is because the sufficiently coherent crystallographic regions of these intermediate *n*‐number phases have not been formed in the (PEA)_2_MA_3_Pb_4_I_13_,^[^ ^29^ ^]^ which is supported by the dominant phases of *n* = 1, *n* = 2, and *n* = ∞ as discussed below.

Steady‐state PL spectroscopy was employed to elucidate the spatial distribution of different *n*‐number phases in the (PEA)_2_MA_3_Pb_4_I_13_. When it is excited from the rear (FTO) side, multiple and broad PL emission peaks across the wavelength region of 500–825 nm are observed, suggesting the existence of multiple phases (Figure S6b, Supporting Information). The strong PL emission peaks at 524, 577, and 770 nm phases again indicate that the *n* = 1, *n* = 2, and *n* = ∞ phases are dominant among others. Differently, when the (PEA)_2_MA_3_Pb_4_I_13_ are excited from the front (perovskite) side, it only shows a PL emission peak of the *n* = ∞ phase. Thus, we conclude that the bottom layer of (PEA)_2_MA_3_Pb_4_I_13_ mainly consists of *n* = 1, *n* = 2, and *n* = ∞ perovskite phases, with low *n*‐number and high *n*‐number phases preferentially accumulated at the bottom and on the top, respectively. Such a gradient distribution of the low‐dimensional perovskite phases is conducive to the directional carrier transport by virtue of the favorably aligned band edges across the (PEA)_2_MA_3_Pb_4_I_13_ layer.^[^ ^13^, ^30^ ^]^ In light of all these results, a schematic diagram is drawn in Figure 1i to illustrate the unique structure of the double‐layer perovskite, which to our surprise has not yet been reported so far. Specifically, the double‐layer perovskite film consists of a bottom layer of gradient 2D perovskite (nominal composition: (PEA)_2_MA_3_Pb_4_I_13_) and a top layer of MAPbI_3_. The gradient 2D perovskite has a layered structure with the low *n* and high *n* phases preferentially accumulated at the bottom and on the top, respectively, while the 3D MAPbI_3_ is crystallized with a preferred vertical orientation.

### 2.2 From Double Layer Perovskite Films to Detector Design: Photoelectric Properties

**Figure 2:**
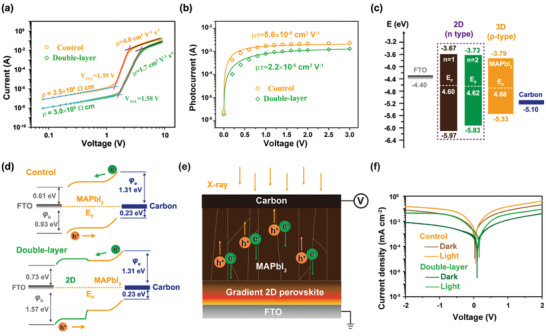
a) SCLC measurements of the detector with a device structure of FTO/Peorvskite/Carbon. b) Photoconductivity measurements of the control and double‐layer perovskite detectors, illuminated by a 532 nm LED (1 mW cm^−2^); c) Schematic diagram showing band edge positions of the 2D perovskites and the 3D MAPbI_3_ with respect to FTO and carbon. d) Interfacial energy level alignment in the devices made of the control and double‐layer perovskite films. e) Device configuration of the double‐layer perovskite X‐ray detectors: the carbon electrode is exposed to the incident X‐ray photons and negatively biased, while the gradient 2D perovskite is prepared by a precursor solution with a nominal composition of (PEA)_2_MA_3_Pb_4_I_13_. f) *J–V* characteristics of the X‐ray detectors made of the control and the double‐layer perovskite films.

To elucidate the impact of the 2D (PEA)_2_MA_3_Pb_4_I_13_ on the optoelectronic properties of the double‐layer perovskite, time‐resolved photoluminesence (TRPL) spectroscopy was carried out. As shown in the Figure S7 (Supporting Information), the control and double‐layer perovskites exhibit similar carrier lifetime of about 49 ns, which is comparable to that reported for polycrystalline MAPbI_3_.^[^ ^31^ ^]^ The almost identical carrier lifetime implies a similar quality between the 3D perovskite films in the control sample and the double‐layer perovskite sample. Considering the fact that the TRPL results merely manifest the interfacial carrier kinetics with no reliable information about the bulk carrier kinetics, we carried out space‐charge‐limited current (SCLC) and photoconductivity measurements to accurately investigate the carrier transport property of the control and double‐layer perovskites. The SCLC measurements were performed on the device structure of FTO/Perovskite/Carbon (Figure S8, Supporting Information). As shown in **Figure** **2**a, three distinctive regions, namely ohmic, trap‐filled limit and Child's regions, are clearly distinguished. By fitting the curves in the Child region, carrier mobility (*μ*) of the control and double‐layer perovskites is calculated to be 4.0 cm^2^ V^−1^ s^−1^ and 1.7 cm^2^ V^−1^ s^−1^, respectively. Plausibly, this decreased mobility is a result of the intrinsically poorer carrier transport of the 2D perovskite, which to some extent retards the charge collection. On the bright side, however, the presence of 2D perovskite brings forth a much higher film resistivity (*ρ*) of 3.0 × 10^9^ Ω cm than that of the control (3.5 × 10^8^ Ω cm), which squarely serves our purpose for reducing the dark current. Knowing the film resistivity and the carrier mobility, the intrinsic carrier concentration (*N*_c_) can be calculated from *N*_c_ = 1/*eμρ*,^[^ ^6c^ ^]^ which are 4.5 × 10^9^ and 1.2 × 10^9^ cm^−3^ for the control and double‐layer perovskite, respectively. Evidently, besides the reduced mobility, the decreased intrinsic carrier concentration also contributes to the enlarged film resistivity of the double‐layer perovskite. Most probably, the presence of the 2D perovskite reduces the defect density (from 2.3 × 10^13^ to 2.1 × 10^13^ cm^−3^) or suppresses the ion conduction.

Although the mobility is slightly sacrificed by introducing the 2D perovskite, we show below that the overall charge collection is not compromised. Figure 2b shows the photoconductivity measurement results of the detectors made of the control and double‐layer perovskite. One can extract *µτ* product by fitting the voltage‐dependent photocurrent to a simplified Hecht equation^[^ ^3a^ ^]^

(1) $$I\;=I_{0}\;\frac{\mu \tau V}{L^{2}}\left(1-\exp\left(-\frac{L^{2}}{\mu \tau V}\right)\right)$$

where *I*_0_ is the saturated current, *L* is the thickness of the perovskite, and *V* is the applied voltage. The *µτ* products of the control and double‐layer perovskites are determined to be 5.6 × 10^−5^ and 2.2 × 10^−5^ cm^2^ V^−1^, respectively, which follow the same trend of the carrier mobilities derived from the SCLC measurements. With the *µτ* product and the electric field *E*, we can get the Schubweg distance *µτE*, which is more directly related to charge collection since it determines the average distance carriers travel before getting trapped or recombined. Generally, for efficient charge collection, an X‐ray absorber should be designed with a thickness less than the Schubweg distance.^[^ ^3b^ ^]^ Assuming a voltage of 2 V is applied to the 60 *μ*m thick perovskite in our case, the Schubweg distances of the control and the double‐layer perovskite are determined to be 184.5 and 72.5 *μ*m, respectively, both of which are larger than their thicknesses and thus can ensure efficient carrier extraction.

Next, we conducted ultraviolet photoemission spectroscopy (UPS) and UV–vis absorption spectroscopy to study the interfacial energy alignment between the 2D and 3D perovskites as well as between the double‐layer perovskites and a given carrier transport layer or charge collection layer, which need to be installed when specific optoelectronic devices are to be made from these materials (Figures S9 and S10, Supporting Information). As shown in Figure 2c, the conduction band minimum (CBM) of the 2D perovskite (−3.67 eV for *n* = 1 and −3.73 eV for *n* = 2) resembles that of the 3D MAPbI_3_ (−3.79 eV). However, substantial difference is found in the valence band maximum (VBM), with −5.97 eV for *n* = 1, −5.83 eV for *n* = 2, and ‐5.33 eV for MAPbI_3_. This is understandable since the interaction of I (5p) with Pb (6s) is affected much more than with Pb (6p) by quantum confinement due to the closer energies of the former pair. The different band structures of the 2D and 3D perovskites will have a direct influence on the detector and functioning as will be shown below. Now by further taking the work function (*W*_F_)/Fermi level (*E*_F_) into account, we can construct the energy band diagrams of the control and double‐layer perovskite devices as depicted in Figure 2d. In the device made of a control film, due to the lower *E_F_* of MAPbI_3_ than that of FTO (−4.68 eV vs −4.40 eV), when bringing them into contact, the *E*_F_ of MAPbI_3_ will line up leading to downward band‐bending at the interface. Conversely, upward band‐bending will result at the MAPbI_3_/Carbon interface. Thus, the control device should be operated with carbon electrode being negatively biased, and the Schottky barriers for hole injection (*φ*_h_) and electron injection (*φ*_e_) are found to be 0.93 and 1.31 eV, respectively, at the two charge collection interfaces. In the double‐layer perovskite device, however, since the 2D perovskite has a deeper VBM, the *φ*_h_ for hole injection is markedly enlarged to 1.57 eV, while the carrier extraction is little affected due to the favorable band alignment between the 2D and 3D perovskites. Notably, the suppression of hole injection stemming from the enlarged *φ*_h_ is expected to significantly reduce the dark current, thus favorably enhancing the SNR.

### 2.3 X‐Ray Detector Performance Assessment

**Figure** 2e shows the device configuration for direct conversion X‐ray detection based on the double‐layer perovskite films. Carbon was used as the top electrode for its low cost and negligible X‐ray attenuation, through which the incident X‐ray photons entered. In consideration of the aforementioned interfacial energy level alignment in the detectors, the carbon electrode is negatively biased to promote carrier extraction. The current density (*J*)–voltage (*V*) characteristics are compared with those of the detector made of the 3D MAPbI_3_ (control device), as shown in Figure 2f. First of all, the double‐layer perovskite detector shows obviously reduced dark current and much increased on–off ratio, indicative of overall enhanced performance for X‐ray detection. Besides, the control and double‐layer perovskite devices show a minimum current at 32 and 99 mV in the dark. Generally, such a voltage shift originates from a non‐strict dark condition, ion migration or localized fields.^[^ ^32^ ^]^ Considering that the two devices are measured under the same condition and the suppressed ion migration in double‐layer perovskite is validated beyond doubt, which will be discussed later, the voltage shift of the double‐layer perovskite device is thus attributed to a localized field associated with the 2D perovskite. Similar phenomenon has been reported previously when the surface of a 3D perovskite is treated by PEABr.^[^ ^32b^ ^]^ Upon exposure to X‐ray irradiation, the current minimum is shifted to 55 mV for the control and 156 mV for the double‐layer perovskite, suggesting the existence of a larger built‐in potential in the double‐layer perovskite. This slightly enlarged built‐in potential probably results from the interfacial energy level alignment induced by the 2D perovskite interlayer, which promotes the carrier extraction in the device.

**Figure 3:**
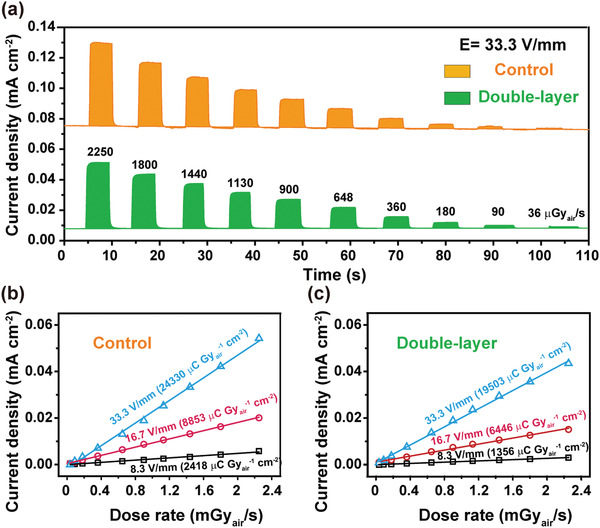
a) The X‐ray response profiles of the control and the double‐layer perovskite detectors with a varying dose rate ranging from 2.25 mGy_air_ s^−1^ to 36 *μ*Gy_air_ s^−1^; The X‐ray generated current density as a function of dose rate at different applied electrical fields for b) the control detector and c) the double‐layer perovskite detector.

To gain dynamic information of the detectors, time‐resolved current density profiles of the detectors under X‐ray exposure with dose rate varying from 2.25 mGy_air_ s^−1^ to 36 *μ*Gy_air_ s^−1^ were recorded and presented in **Figure** **3**a. It is found that the control and double‐layer perovskite detectors both exhibit decent response signals upon on/off switching of X‐ray. More importantly, through detailed analysis on the response characteristics of the detectors exposed to X‐ray at a dose rate of 2.25 mGy_air_ s^−1^ (Figure S11, Supporting Information), we observe that the presence of 2D perovskite does not slow down the response speed (≈200 ms), and yet it considerably reduces the dark current density (*J*_D_) from 75.5 to 7.7 µA cm^−2^. Clearly, the large dark current of the control is due to the low resistivity of MAPbI_3_ (3.5 × 10^8^ Ω cm), high electrical filed (33 V mm^−1^), and possible carrier injection from the electrode. Then naturally, the reduction in *J*_D_ with the presence of 2D perovskite stems from the improved film resistivity and enlarged hole injection barrier. Significantly, the reduced dark current makes the double‐layer perovskite detector more easily integrated to the existing electronic readout technologies such as thin film transistor arrays, wherein the capacitors, used for signal storage, generally have a limited capacitance.^[^ ^33^ ^]^ Similarly, the photocurrent density (*J*_P_) also drops from 129.7 to 51.1 µA cm^−2^, which in turn gives rise to an X‐ray generated current density (*J*_S_ = *J*_P_ − *J*_D_) of 54.2 and 43.5 µA cm^−2^ for the control and double‐layer perovskite, respectively. This slightly decreased *J*_S_, but remarkably reduced *J*_D_, implies that the detector made of the double‐layer perovskite could achieve a much lower LoD while offering a competitive sensitivity as compared to the MAPbI_3_ counterpart.

More insight can be obtained from the following analysis. To start with, the theoretical X‐ray generated current density (*J*_T_) is estimated to be 3.91 nA cm^−2^ according to the following equations^[^ ^8^, ^20^, ^34^ ^]^

(2) $$J_{T}=\varphi \beta e/A$$

(3) $$\varphi =\varepsilon Dm_{s}/E_{\mathrm{ph}}$$

(4) $$\varepsilon =1-\exp\left(-\alpha L\right)$$

(5) $$\beta =E_{\mathrm{ph}}/W_{\pm }$$

(6) $$W_{\pm }=2E_{g}+1.43$$

(7) $$G=J_{S}/J_{T}$$

where *φ* is the photon absorption rate, *β* is the maximum number of carriers excited by an X‐ray photon, *e* is the elementary charge, *A* is the active area, *ε* is the attenuation ratio (Figure S12, Supporting Information), *D* is the dose rate, *m*_s_ is the sample mass, *E*_ph_ is the photon energy (40 keV), *α* is the attenuation coefficient, *W*_±_ is ionization energy, and *E*_g_ is the bandgap. Thus, the gain factor (*G*) is determined to be 1.39 × 10^4^ for the control and 1.11 × 10^4^ for the double‐layer perovskite. To discriminate between contributions from hole and electron reinjection to the photoconductive gain, we fabricated the detectors of FTO/double‐layer perovskite/poly(triarylamine) (PTAA)/carbon and FTO/TiO_2_/double‐layer perovskite/carbon, where PTAA blocks electron reinjection from carbon and TiO_2_ blocks hole reinjection from FTO (Figure S13a,b, Supporting Information). It is found that with the presence of TiO_2_, the first detector exhibits obviously decreased photoconductive gain, whereas the X‐ray response of the PTAA inserted detector (the second detector) resembles that of FTO/double‐layer perovskite/carbon without PTAA inserted (Figure S13c,d, Supporting Information). These results suggest that the photoconductive gain in the double‐layer perovskite is mainly due to hole recycling, consistent with the p‐type MAPbI_3_ used in our case (Figure 2c).

It has been previously hypothesized that efficient hole recycling under illumination can be enabled by electron trapping at the interfaces, and this is despite a large hole injection barrier in the dark since the electron trapping at the interfaces can modify interfacial band bending thereby reducing the Schottky junction thickness and barrier for hole injection from the electrode.^[^ ^35^ ^]^ To validate this hypothesis, we fabricated a detector made of double‐layer perovskite with a 4.1 *μ*m thick (PEA)_2_MA_3_Pb_4_I_13_ film, which was intended to suppress hole tunneling from the positively biased FTO electrode.^[^ ^36^ ^]^ As shown in Figure S15 (Supporting Information), the detector exhibits a substantially decreased photoconductive gain of 286 at 10 V mm^−1^, comparable to that of the detector adopting TiO_2_ as the hole injection blocking layer (Figure S13b,d, Supporting Information), demonstrating the effectiveness of the 4.1 *μ*m thick (PEA)_2_MA_3_Pb_4_I_13_ in suppressing the hole recycling channels. This observation suggests that a large thickness of (PEA)_2_MA_3_Pb_4_I_13_ appears to be detrimental to the sensitivity of the X‐ray detectors.

To quantify the sensitivity of the control and double‐layer perovskite detectors, the X‐ray generated current density as a function of the dose rate is extracted. As shown in Figure 3b,c, the photocurrent shows a good linear relationship with the dose rate ranging from 36 *μ*Gy_air_ s^−1^ to 2.25 mGy_air_ s^−1^, and the sensitivity is derived from the slope of the photocurrents versus X‐ray dose rates. For the control detector at reverse electrical fields of 8.3, 16.7, and 33.3 V mm^−1^, the sensitivities are 2418, 8853, and 2.43 × 10^4^ µC Gy_air_^−1^ cm^−2^, respectively, which are much higher than those of ɑ‐Se (20 µC Gy_air_^−1^ cm^−2^ at 1000 V mm^−1^).^[^ ^3c^ ^]^ The electrical field‐dependent sensitivity is attributed to the field‐enhanced carrier reinjection: the larger the electrical bias, the higher the photoconductive gain.^[^ ^7^, ^8^ ^]^ Remarkably, with insertion of the much more resistive 2D perovskite layer, the double‐layer perovskite detector exhibit only a slightly compromised sensitivity of 1.95 × 10^4^ µC Gy_air_^−1^ cm^−2^ at 33.3 V mm^−1^, which outperforms most MAPbI_3_ X‐ray detectors reported to date (Table S1, Supporting Information).

On the other hand, the detectors adopting a mixed‐dimensional perovskite (nominal composition: (PEA)_2_MA_3_Pb_4_I_13_) and a 2D/3D/2D sandwich perovskite were also fabricated for comparison (Figures S16 and S17, Supporting Information). In stark contrast to the double‐layer perovskite, the 2D/3D mixed perovskite (nominal composition: (PEA)_2_MA_3_Pb_4_I_13_) exhibits obvious diffraction peaks at low angles (2*θ* < 14°), suggesting the existence of multiple low‐dimensional phases in the bulk (Figure S16c, Supporting Information). However, due to the lack of a gradient distribution of the low‐dimensional perovskite phases in the vertical direction, the carrier transport in the 2D/3D mixed perovskite is expected to be severely impeded by the unfavorably aligned band edges. As a result, the 2D/3D mixed perovskite only delivers a sensitivity of 153 *μ*C Gy_air_^−1^ cm^−2^, which is 127 times lower than that of the double‐layer perovskite with the advantageous 2D/3D perovskite gradient distribution and orientation. Turning to the 2D/3D/2D sandwich perovskite detector, we get an even lower dark current of 0.12 *μ*A cm^−2^, as well as a quite stable baseline. However, the 2D/3D/2D perovskite detector suffers from its rather slow X‐ray response at low electrical fields (<25 V mm^−1^), contrary to the double‐layer perovskite operated at similar electrical fields (Figure S18, Supporting Information), indicating that the charge collection is significantly impeded. Although this retarded X‐ray response can be alleviated by applying a high electrical field, one can only get a low sensitivity of 130.4 *μ*C Gy_air_^−1^ cm^−2^ (Figure S17f, Supporting Information), which is 150 times lower than that of the double‐layer perovskite. As demonstrated above, the high sensitivity of double‐layer perovskite detector stems from the efficient hole recycling (Figure S13, Supporting Information). However, in the presence of the 2D perovskite on top, efficient hole transport cannot be maintained due to the unfavorable interfacial energy level alignment (Figure S19, Supporting Information) and the further decreased carrier mobility. Therefore, we conclude that although the 2D/3D/2D perovskite is advantageous in achieving a low dark current, it cannot grantee a high sensitivity at the same time due to the lack of a proper interfacial energy level alignment.

**Figure 4:**
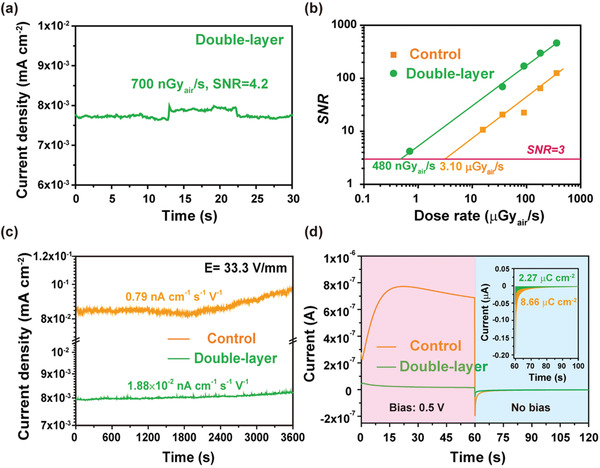
a) The X‐ray response of the double‐layer perovskite detector at an ultralow dose of 700 nGy_air_ s^−1^; b) The dose rate dependent SNR of the control and the double‐layer perovskite detector; c) The temporal baseline tracking of the detectors made of the control and the double‐layer perovskite film; d) The temporal current profiles of the control and double‐layer perovskite detector under the bias and no bias conditions (the inset enlarges the no bias portion and shows the corresponding time integrated areal charge densities). In (d), the detector was first subjected to a bias to drive migration of the ions to the interfaces, followed by relaxation of the accumulated ions.

Besides, the SNR at different dose rate is calculated to evaluate the LoD of the detectors according to the equations of

(8) $$\mathrm{SNR}=J_{s}/J_{n}$$

(9) $$J_{n}=\sqrt{\frac{1}{N}\sum_{i}^{N}{\left(J_{i}-J_{p}\right)}^{2}}$$

where *J*_s_ is the X‐ray generated current density, and *J*_n_ is the noise current density which is the standard deviation of the photocurrent density (*J*_p_). **Figure** **4**a shows the X‐ray response of the double‐layer perovskite detector with an ultralow dose rate of 700 nGy_air_ s^−1^, where an SNR of 4.2 is derived, validating its strong capacity for low‐dose X‐ray detection. Furthermore, the SNR of the detectors as a function of dose rate is plotted in Figure 4b. Evidently, the SNR of the detectors shows a nice linear dependence with dose rate, thus the LoD can be obtained by extrapolating the SNR to the value of 3.^[^ ^12b^ ^]^ As expected, with the 2D perovskite layer, the LoD is obviously reduced from 3.12 *μ*Gy_air_ s^−1^ for the control detector to 480 nGy_air_ s^−1^ for the double‐layer perovskite detector. To be sure, this LoD, even much reduced, still cannot compete with that of the 0D lead‐free perovskite (0.62–130 nGy_air_ s^−1^) due to the orders of magnitude higher resistivity (2.8 × 10^10^–2.3 × 10^11^ Ω cm) of the latter, highlighting the need to further increase the film resistivity.^[^ ^10^, ^11^, ^12^, ^37^ ^]^ Nonetheless, the LoD is an order of magnitude lower than that of the commercial ɑ‐Se detectors (5.5 *μ*Gy_air_ s^−1^), and thus can meet the general requirements for medical imaging.^[^ ^34b^ ^]^

In addition to high sensitivity and low LoD, a stable baseline is another indispensable prerequisite for high performance X‐ray detectors, which need to avoid the overflow error and ensure accurate signal recording.^[^ ^38^ ^]^ In this connection, we tracked the temporal baseline evolution of the detectors at 33.3 V mm^−1^ for 1 h. As shown in Figure 4c, the severe baseline drifting observed for the control detector (0.79 nA cm^−1^ s^−1^ V^−1^) was drastically reduced for the double‐layer perovskite detector (0.019 nA cm^−1^ s^−1^ V^−1^), clearly due to the mitigation of ion migration in the 2D perovskite.^[^ ^20^ ^]^ To further verify this scenario, the concentrations of the mobile ions in the detectors were estimated by first applying a bias to drive the mobile ions to the interfaces, followed by relaxation of the accumulated ions.^[^ ^39^ ^]^ As shown in Figure 4d, once the bias is removed, the detector immediately shows a negative ionic current. By integrating the ionic current, it is found that the doulbe‐layer perovskite detector has a much lower charge density (*D*) of 2.27 *μ*C cm^−2^ than the control detector (8.66 *μ*C cm^−2^). From these data, the mobile ion concentrations of the control and double‐layer perovskite films are estimated to be 3.7 × 10^16^ and 8.9 × 10^15^ cm^−3^, respectively, according to the equation *N* = *D*/*eL*, where *N* is the mobile ion concentration, *e* is the elementary charge and *L* is the thickness.^[^ ^39^ ^]^

**Figure 5:**
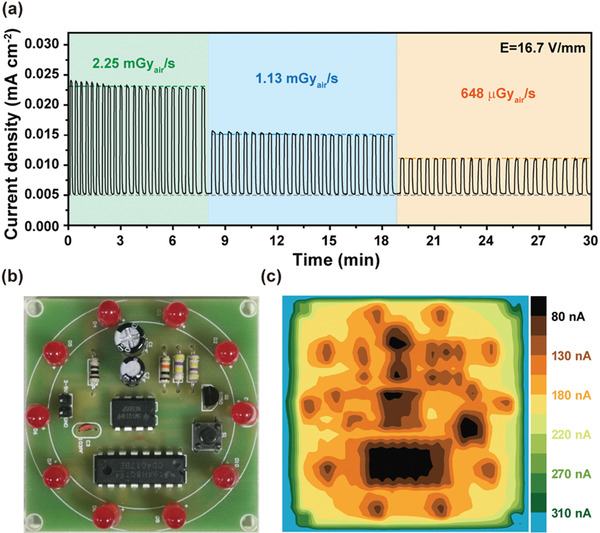
a) Stability test of the double‐layer perovskite detector at different dose rates of X‐ray exposure; b) A digital photograph of the printed circuit board (size: 5.1 cm × 5.1 cm); c) An X‐ray image of the printed circuit board acquired with the double‐layer perovskite detector with an active area of 3 mm × 3 mm (*E* = 16.7 V mm^−1^, Dose rate: 0.4 mGy_air_ s^−1^).

Lastly, the potential of the double‐layer perovskite detector for practical use is demonstrated by testing the device stability and its X‐ray imaging capacity. As shown in **Figure** **5**a, the double‐layer perovskite detector shows a highly reproducible X‐ray response upon periodical on/off switching of the X‐ray. With such decent device stability, we further evaluated its X‐ray imaging capacity with a home‐built *x*–*y* scanning system. The X‐ray image detector was intentionally designed with a moderate active area of 3 mm × 3 mm to simultaneously ensure acceptable device area error, scanning accuracy, and image resolution, and a printed circuit board (PCB) was selected as the imaging target. As shown in Figure 5b,c, the main outline of the PCB is successfully distinguished at a low dose rate of 0.4 mGy_air_ s^−1^. In general, the image resolution of an X‐ray imaging system is determined by not only the active layer (perovskite) but also the pixel resolution (size). The relatively low image resolution here is a result of the large pixel size we used, indeed even larger than some of the electronic components on the PCB, for the mere demonstration of principle. Still, we are convinced of the great potential of the high‐quality double‐layer perovskite in direct X‐ray imaging. Moreover, it is anticipated that by further reducing the active area of the detector or integrating the double‐layer perovskite with commercial electronic readout circuits, the image quality will be dramatically improved.

## 3 Conclusion

In summary, by exploiting a sequential deposition technique with high controllability, we have developed a high‐performance X‐ray detector made of a 2D/3D double‐layer perovskite film, where the layered 2D (PEA)_2_MA_3_Pb_4_I_13_ is cascaded with a vertical crystallized 3D MAPbI_3_. The 2D perovskite greatly suppresses the ion migration, thus stabilizing the baseline of the double‐layer perovskite detectors and timely addressing the baseline drifting issue that has plagued the community. In addition, with the aligned interfacial energy level and improved film resistivity, the detectors offers a high sensitivity of 1.95 × 10^4^ *μ*C Gy_air_^−1^ cm^−2^ and a low LoD of 480 nGy_air_ s^−1^, which outperforms most MAPbI_3_ detectors reported so far. This work provides a novel strategy to unlock the limitations arising from the intrinsic properties of a single perovskite for enhanced X‐ray detection by smartly combining perovskites with different dimensionalities.

## 4 Experimental Section

### Materials

Methylammonium iodide (MAI, >99.99%) and 2‐phenylethylammonium iodide (PEAI, >99%) were purchased from GreatCell Solar. Lead iodide (PbI_2_, 98%) was bought from Sinopharm. Organic solvents such as dimethyl sulfoxide (DMSO, AR, 99%) and *N*,*N*‐dimethylformamide (DMF, AR, 99%) were supplied by Aladdin. The carbon paste (Product No. DD‐10), used for carbon electrode preparation, was purchased from New Seaside Science and Trade Co., Ltd (Guangzhou, China). All these chemicals were used as received.

### Device Fabrication

First, 1.25 g of PEAI, 1.19 g of MAI, and 4.61 g of PbI_2_, corresponding to PEA_2_(MA)_3_Pb_4_I_13_, were dissolved in a 20 mL DMF/DMSO mixture solution (DMF:DMSO = 1:1, v/v) to form a 2D perovskite precursor solution. At the same time, 3.34 g of MAI and 9.68 g of PbI_2_ were dissolved in a 30 mL DMF/DMSO mixture solution (DMF:DMSO = 1:1, v/v) to get an MAPbI_3_ precursor solution. For the preparation of 2D/3D double‐layer perovskite, an ALS process was employed (Figure S20, Supporting Information). Briefly, aerosol droplets of the 2D perovskite were first generated from its precursor solution under an ultrasonic nebulizer, and carried to a nozzle by nitrogen. Once these droplets arrived the preheated fluorine‐doped SnO_2_ (FTO) glasses at 140 °C, 2D perovskite was immediately formed due to balanced solvent volatilization and perovskite crystallization. The ALS process was circulated for 8 cycles to obtain a 1.6 *μ*m thick 2D perovskite, followed by annealing at 130 °C for 5 min. Successively, the MAPbI_3_ precursor was placed in another cleaned vessel, and the ALS process was carried out for 400 cycles to deposit a 60 *μ*m thick MAPbI_3_, during which the temperature was set as 130 °C. It is worth mentioning that although the whole process took about 3 h, the perovskite did not show any degradation, which was as a result of the continuous spraying of the perovskite aerosol. Then the as‐obtained double‐layer perovskite was cooled down to room temperature with a slow cooling rate of 1°C min^−1^. Finally, the carbon electrode was doctor bladed onto the double‐layer perovskite with a 0.1 mm thick stainless‐steel mask, forming an active area of 0.64 cm^2^. The as‐bladed carbon electrode was thermal annealed at 100 °C for 10 min. For comparison, the MAPbI_3_ detectors were also prepared according to the above protocol simply without the deposition of the 2D perovskite. All these experiments were conducted in air without any control.

### Materials Characterizations

The morphology of the perovskite was monitored by scanning electron microscope (SEM, JEOL 7100F). The UV–vis absorption spectra were obtained on a UV–vis spectrometer (UV‐1800, Shimadzu). The steady state PL spectra and in situ PL spectra were acquired by a PL spectrometer (LTF‐3000, QSpec, Biaoqi, Guangzhou) with a 405 nm laser as the excitation source. During the in situ PL measurement, a sprayed wet (PEA)_2_MA_3_Pb_4_I_13_ film was placed on a hotplate (140 °C), and the PL spectra were automatically recorded every 100 ms. The time‐resolved photoluminescence (TRPL) spectra were recorded by a time‐correlated single‐photon counting spectrometer (Horiba, FluoroMax‐4) with a 450 nm laser as the excitation source. The crystal structure was investigated by acquiring the XRD patterns (D8 Discover, Bruker). The photoconductivity of the control and double‐layer perovskite detectors with a thickness of 60 *μ*m was measured under illumination of a 532 nm LED with a power density of 1 mW cm^−2^, the photo‐induced current density as a function of the voltage was recorded by a Keithley 2612B source meter. The Fermi level and valance band of the perovskite were studied by UPS (ESCALAB 250Xi, Thermo Fisher). The SCLC measurement was performed on a Keithley 2612B source meter. The carrier mobility of the perovskite was deduced by fitting the curves in the Child region with the Mott‐Gurney law of *J* = 9*εε*_o_ *μV* ^2^/8*L* ^3^, where *J* is the current density, *μ* is the carrier mobility, *V* is the voltage bias, *ε* is the relative dielectric constant,^[^ ^40^ ^]^ *ε*_o_ is the permittivity of vacuum, and *L* is the film thickness. The trap density was calculated according to the equation of *V*_TFL_ = *en_t_L* ^2^/2*εε*_o_, where *V*_TFL_ is the onset voltage of trap‐filled limit and *e* is electric charge constant. The transient ionic current was also measured on a Keithley 2612B source meter. An external reverse bias of 0.5 V was applied to the detector (perovskite thickness: ≈15 *μ*m) for 60 s in dark, followed by tracking the transient current for another 60 s at 0 V.

### Device Characterization

The X‐ray detection performance was evaluated by an X‐ray generation system (Varex, G242, 18932‐M8, USA) used for medical imaging. The acceleration voltage was set as 70 kV, the current was varied from 0.2 to 12.5 mA, and a 2 cm thick Al as used as attenuator when necessary. The dose rate of the X‐ray was strictly calibrated by using an X2 CT dosimeter (Unfors Raysafe, Sweden). During the measurement, the detectors were placed in dark, and the external electrical bias and current were recorded by a Keithley 2612B source meter. The X‐ray imaging capacity of the detector was demonstrated by using a home‐built *x*–*y* scanning system to move the investigated object between the detector (9 mm^2^) and the X‐ray beam (0.4 mGy_air_ s^−1^). The home‐built *x*–*y* scanning system consisted of an electric linear displacement platform (Newport, M‐IMS400CC) with a lifting table (Beijing Paidiwei Instrument Co., Ltd.PT‐SD408) fixed on it. The electric linear displacement platform coupled with a motion controller (Newport, M‐IMS400CC) was used to control the scanning along the *x*‐axis, while the lifting table was manually controlled to enable the scanning along *y*‐axis.

## Conflict of Interest

The authors declare no conflict of interest.

## Supporting information

Supporting Information

## Data Availability

The data that support the findings of this study are available from the corresponding author upon reasonable request.
