# Supplementary material for: Sequential Growth of 2D/3D Double‐Layer Perovskite Films with Superior X‐Ray Detection Performance
Source: Adv Sci (Weinh). 2021 Sep 8;8(21):2102730. doi: 10.1002/advs.202102730 (PMC8564448; doi:10.1002/advs.202102730)
Supplement: Supplementary file 1 — Supporting Information [file ADVS-8-2102730-s001.pdf]

© 2021 Wiley-VCH GmbH

ADVANCED SCIENCE
Open Access

Supporting Information

for *Adv. Sci.,* DOI: 10.1002/advs.202002730

Sequential growth of 2D/3D double-layer perovskite films with superior X-ray detection performance

*Xiuwen Xu, Wei Qian, Jian Wang, Jiecheng Yang, Jianwei Chen, Shuang Xiao, Yongshuai Ge, and Shihe Yang**

Supporting Information

Sequential growth of 2D/3D double-layer perovskite films with superior X-ray detection performance

*Xiuwen Xu, Wei Qian, Jian Wang, Jiecheng Yang, Jianwei Chen, Shuang Xiao, Yongshuai Ge, and Shihe Yang**

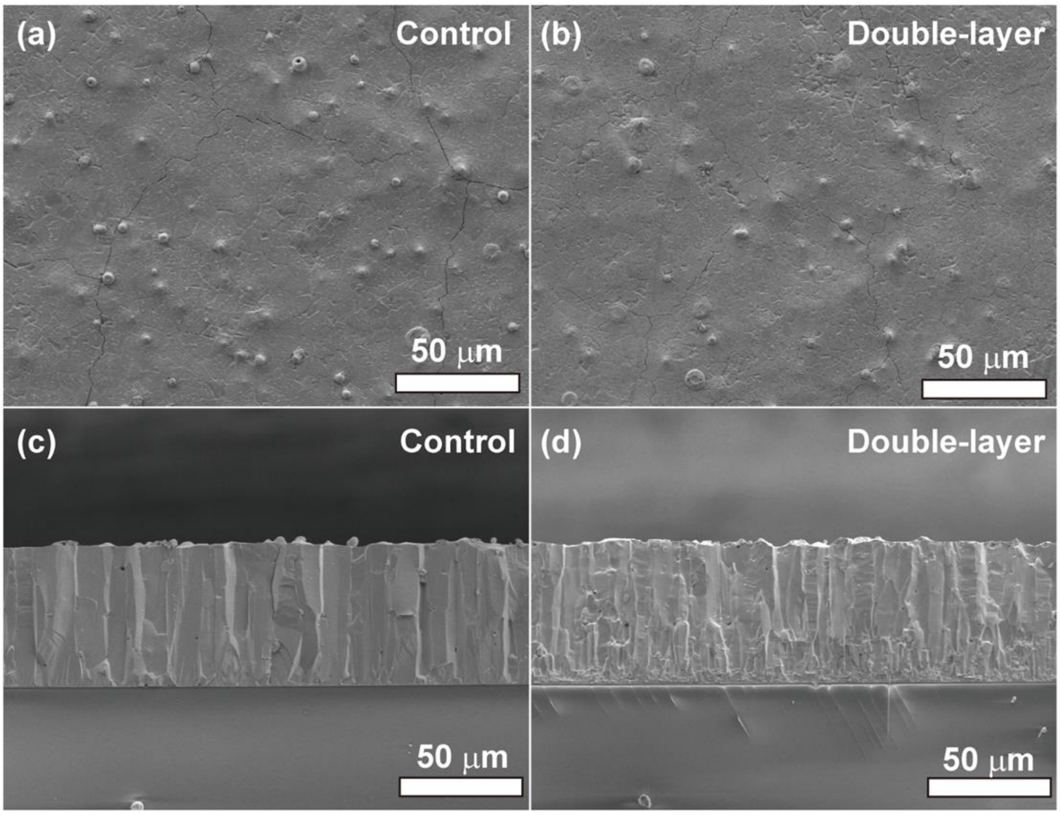

Figure S1. SEM images of the control (a, c) and double-layer perovskite films (b, d).

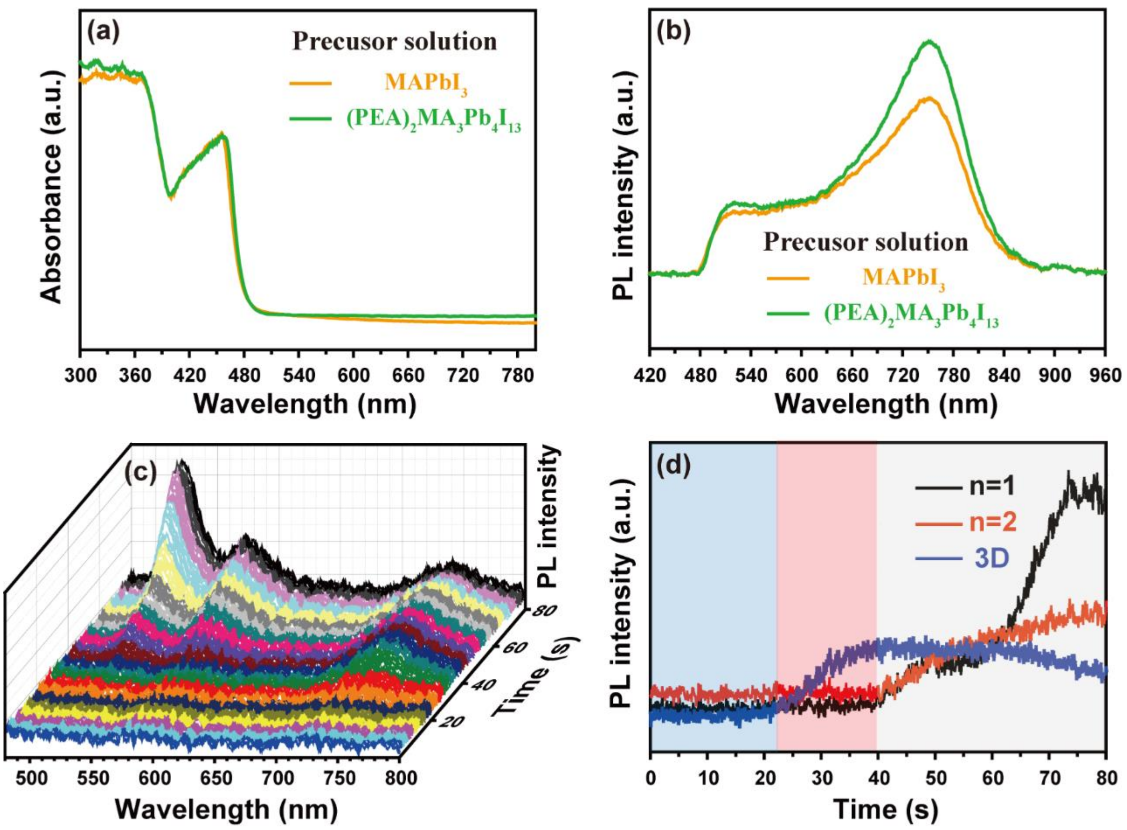

Figure S2. The absorbance (a) and PL spectra of the precursor solution of $MAPbI_3$ and $(PEA)_2MA_3Pb_4I_{13}$; (c) The in-situ PL spectra monitoring the perovskite crystallization in a wet $(PEA)_2MA_3Pb_4I_{13}$ film (nominal composition) that heated at 140 ^{o}C; (d) The temporal evolution of the PL intensity of n=1, n=2 and 3D perovskite, extracted from Figure S2c.

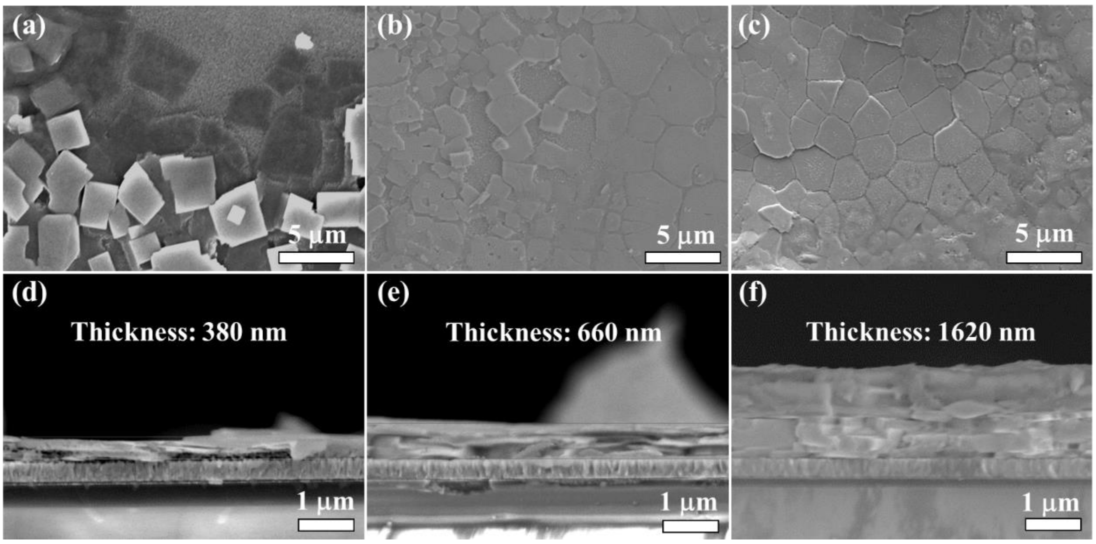

Figure S3. SEM images of the $(PEA)_2MA_3Pb_4I_{13}$ film with different thickness.

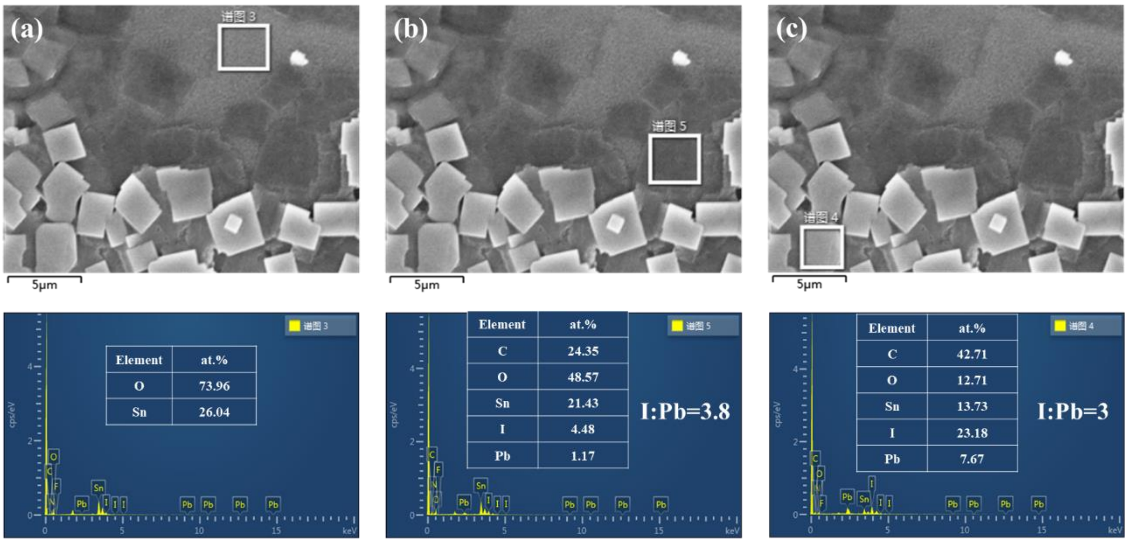

Figure S4. SEM images of the 380-nm-thick $(PEA)_2MA_3Pb_4I_{13}$ film, and the corresponding energy-dispersive X-ray spectra (EDS) of the selected area (indicated by the white rectangle) are shown below.

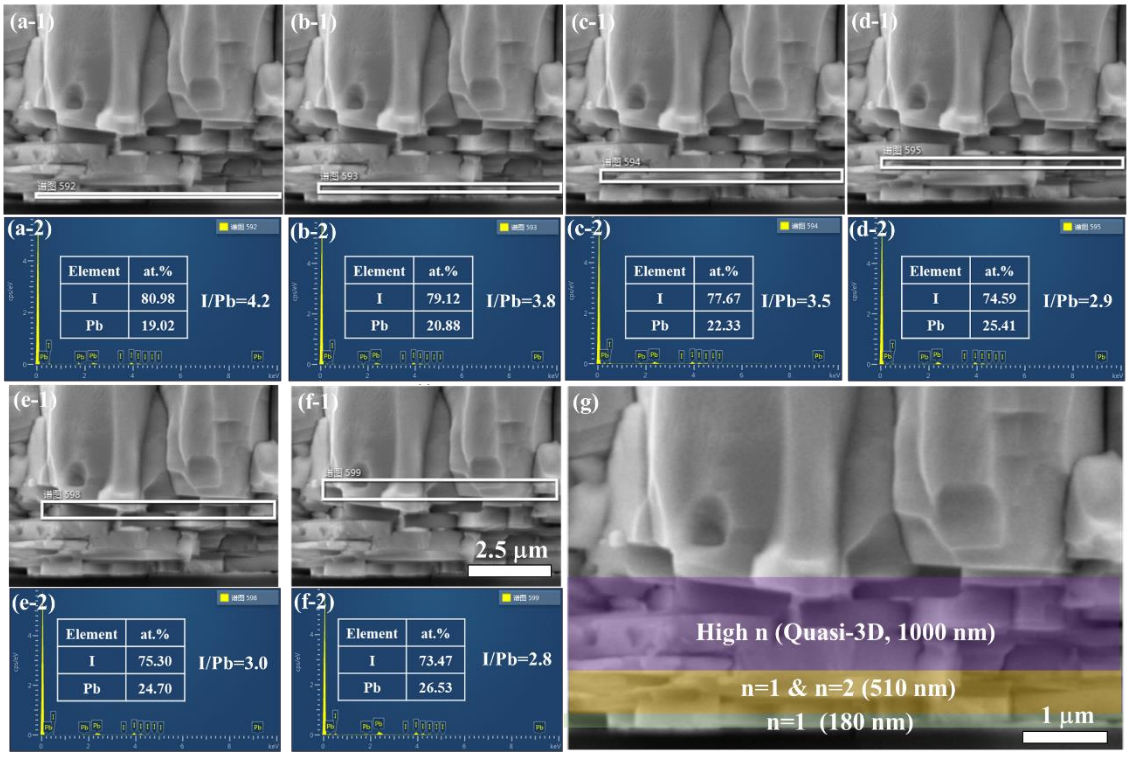

Figure S5. (a-f) Cross-sectional SEM images of the interface of 2D/3D double-layer perovskite and the EDS acquired at different locations indicated by the white rectangles; (g) Illustration of the vertical distribution of the perovskite phases at the interfaces of 2D/3D double-layer perovskite.

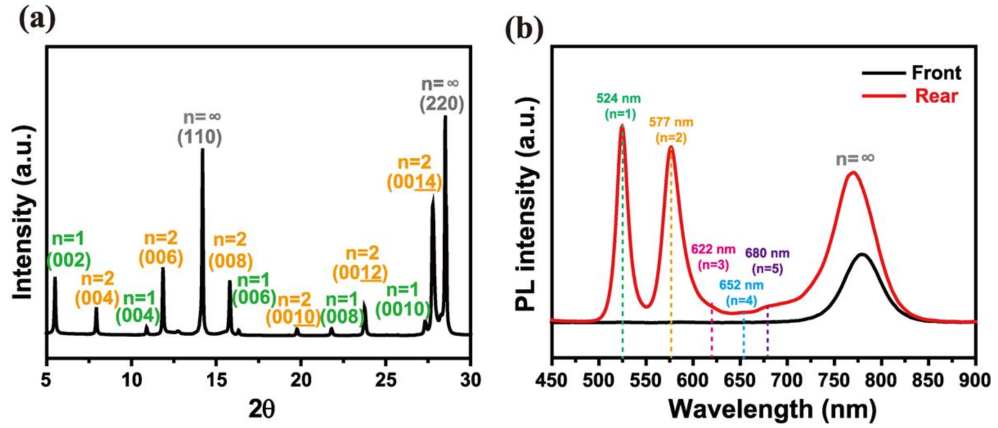

Figure S6. XRD patterns (a) and Steady-state PL spectra (b) of the 1.6-μm-thick $(PEA)_2MA_3Pb_4I_{13}$ film.

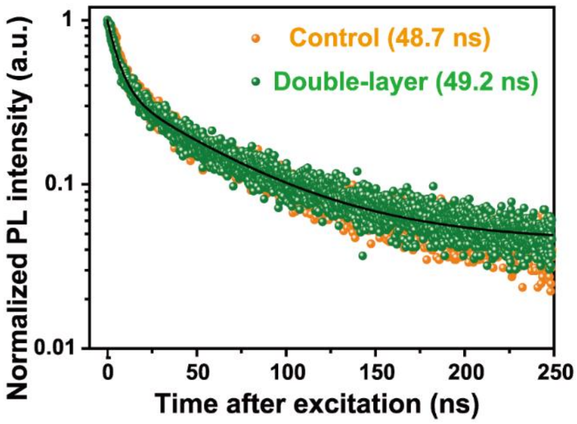

Figure S7. TRPL decay profiles of the control and double-layer perovskite films, excited from the front (Perovskite) side.

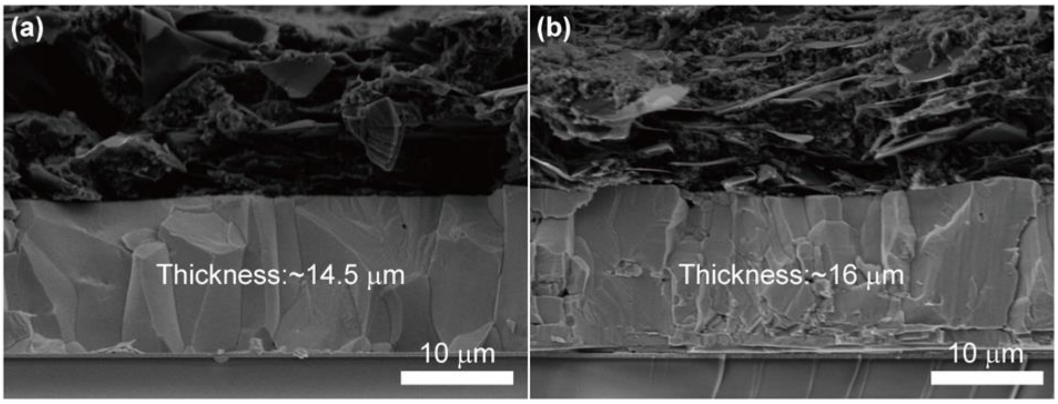

Figure S8. Cross-sectional SEM images of the device made of the control (a) and double-layer perovskite (b) for SCLC and transient ionic current measurements.

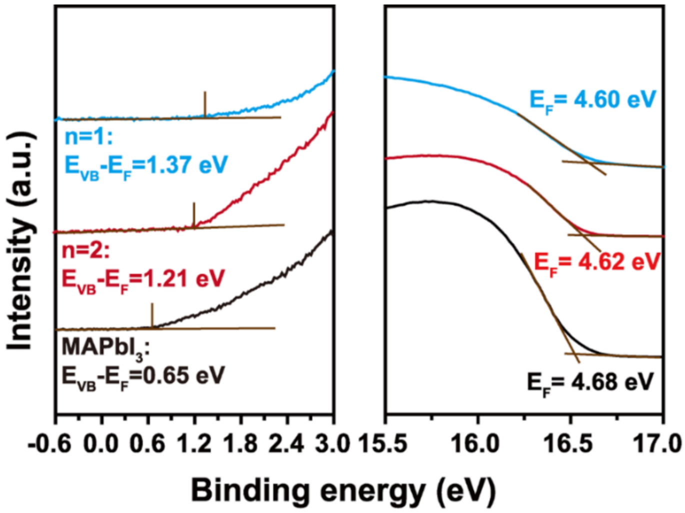

Figure S9. UPS spectra of the $MAPbI_3$ and 2D perovskite of n=1 and n=2 phases.

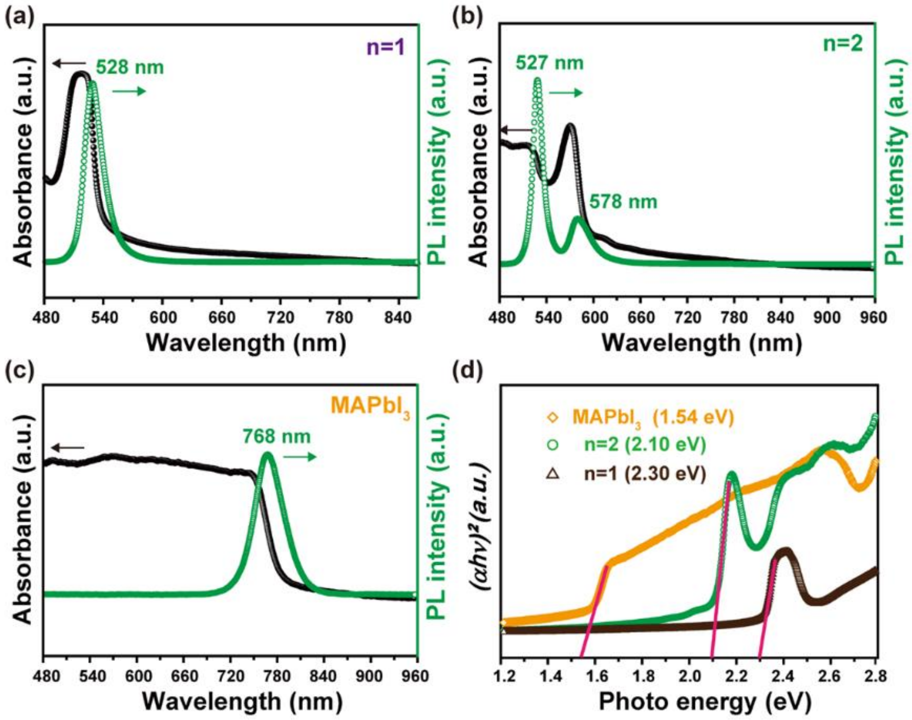

Figure S10. The absorption spectra and steady-state PL spectra of 2D perovskite with n=1 (a) and n=2 phase (b) and $MAPbI_3$ (c); (d) The Tauc plots to estimate the optical band gap.

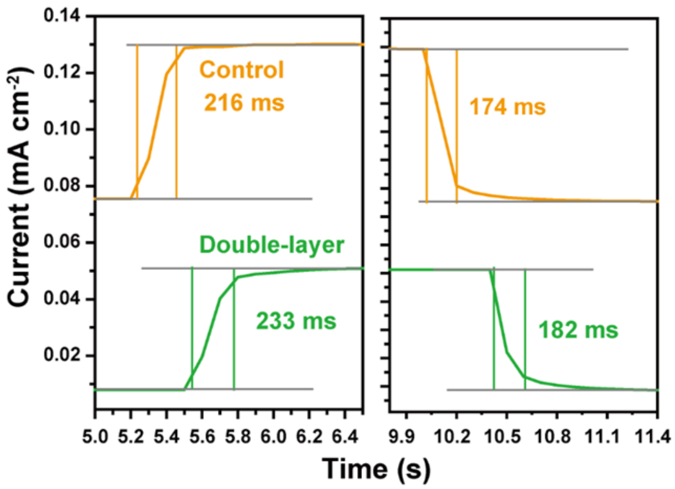

Figure S11. The X-ray response of the detector made of the control and double-layer perovskite, and the applied electrical field is 33.3 V mm^{-1} and the dose rate is 2.25 mGy_{air} s^{-1}.

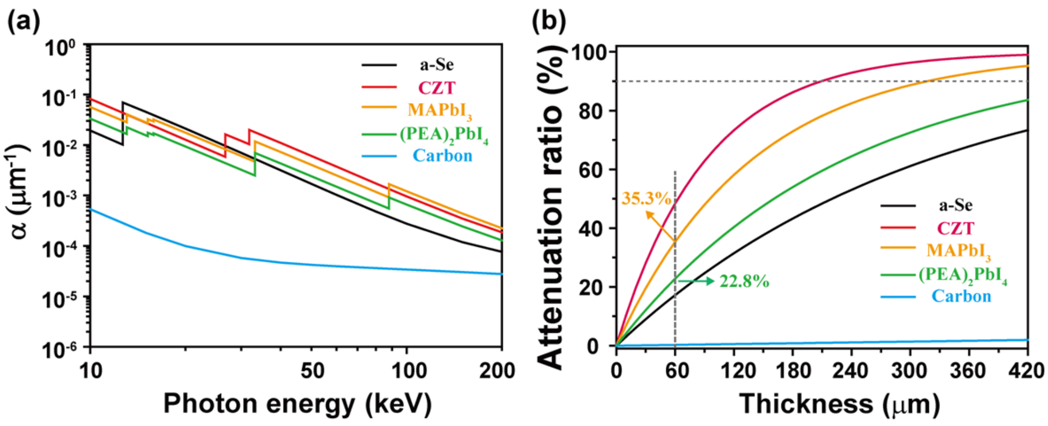

Figure S12. (a) The X-ray attenuation coefficient of different materials; (b) The thickness dependent attenuation ratio of different materials under a 40 keV X-ray irradiation.

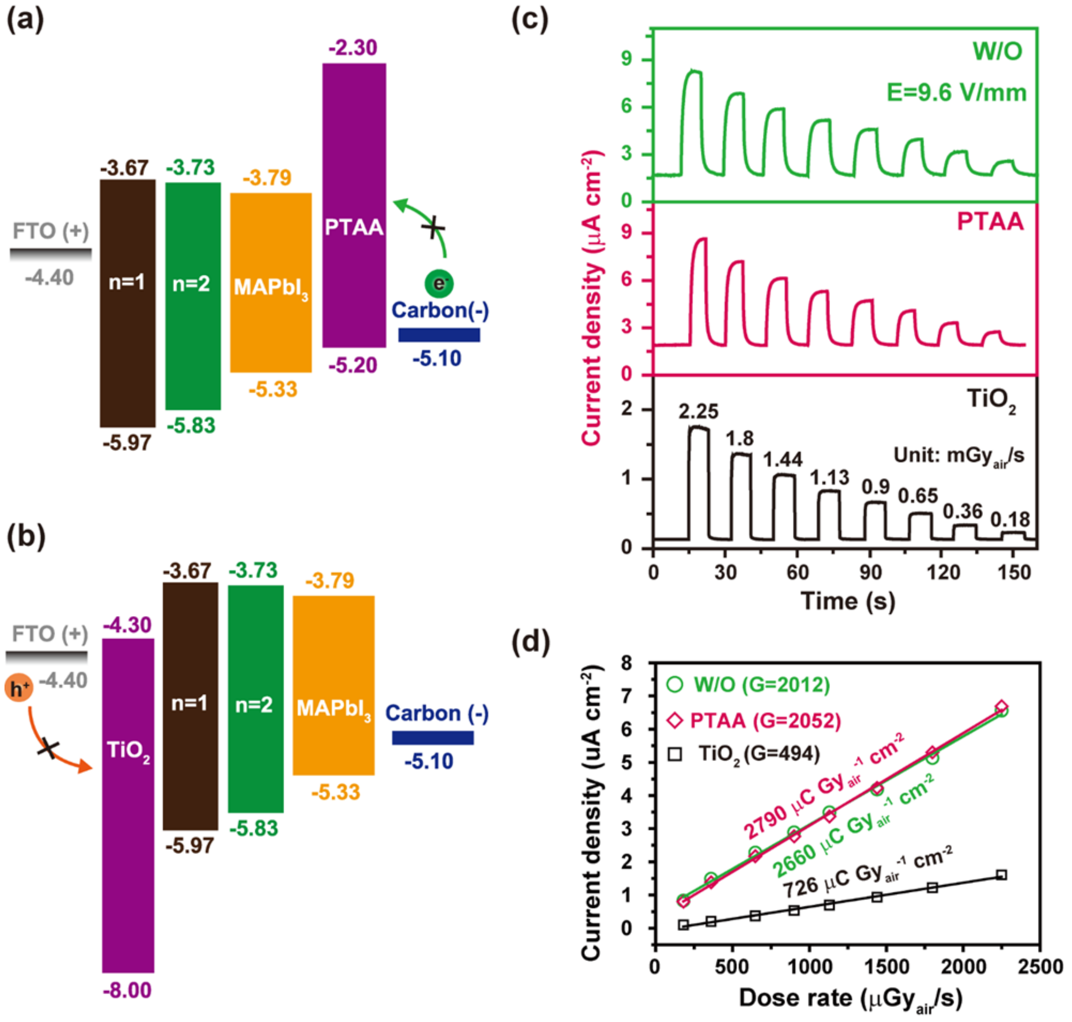

Figure S13. Double-layer perovskite X-ray detector with PTAA as the electron injection blocking layer (a) and with TiO_2 as the hole injection blocking layer (b); (c) X-ray response of the detectors with varied dose rates of X-ray, where FTO is positively biased with 0.5 V, and the thickness of the double-layer perovskite in this batch is 52 μm, as shown in **Figure S14**.

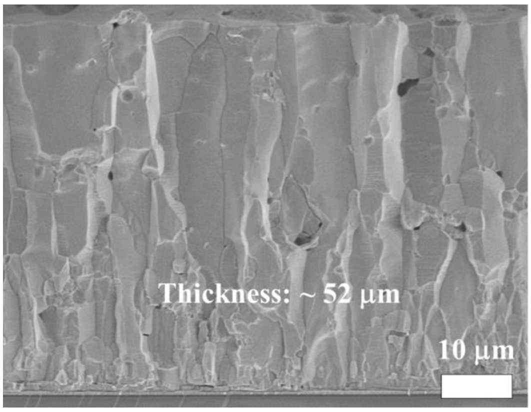

Figure S14. A cross-sectional SEM image of the double-layer perovskite prepared in the batch used for the photoconductive gain investigations.

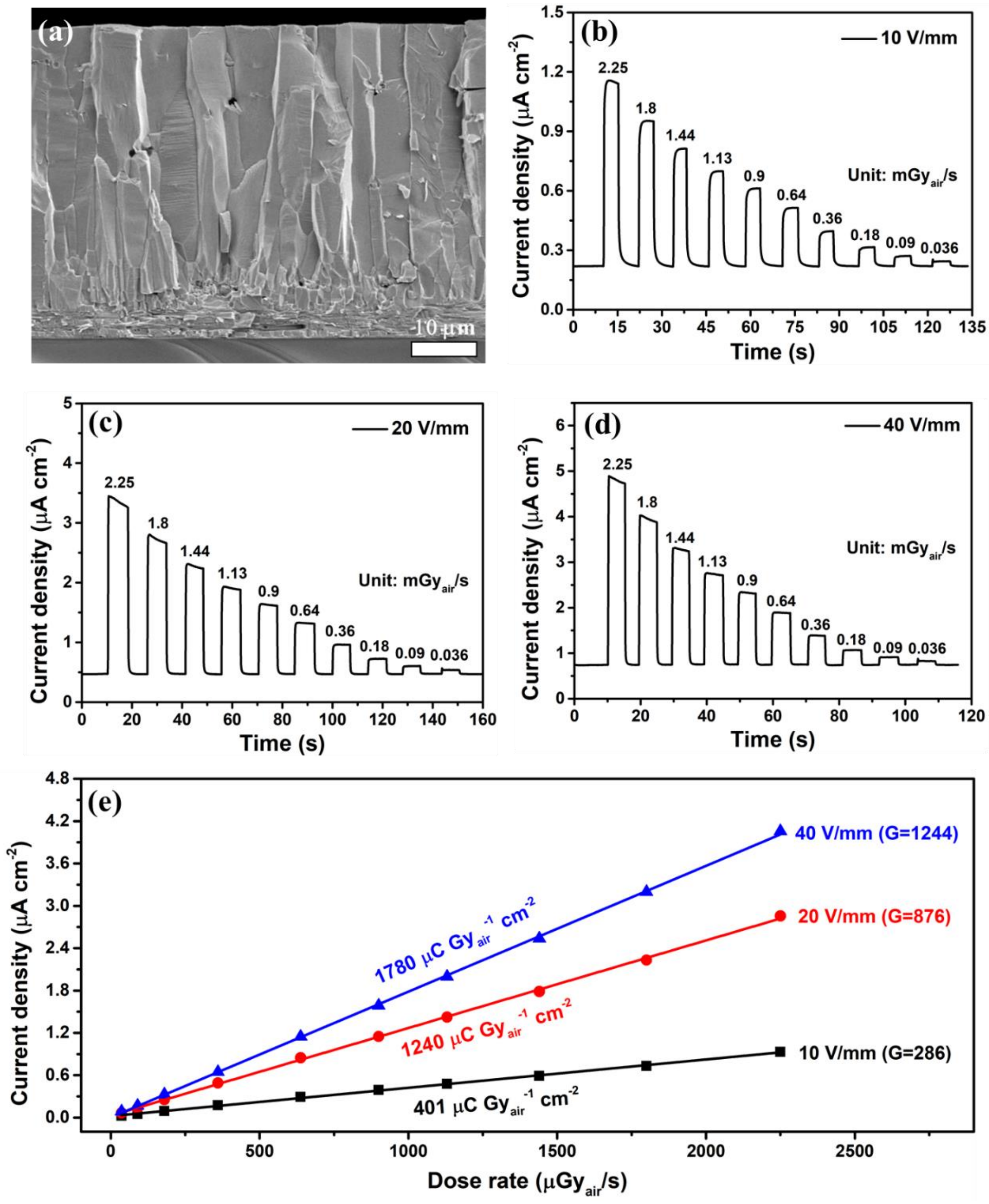

Figure S15. (a) A cross-sectional SEM image of the double-layer perovskite with a 4.1-μm-thick $(PEA)_2MA_3Pb_4I_{13}$ (20 cycles of the ALS process); The X-ray response characteristics of the detector under different electrical felids: (b)10 V/mm; (c) 20 V/mm and (d) 40 V/mm; (f)The electrical field dependent sensitivity of the X-ray detector made of the double-layer perovskite with a 4.1-μm-thick $(PEA)_2MA_3Pb_4I_{13}$.

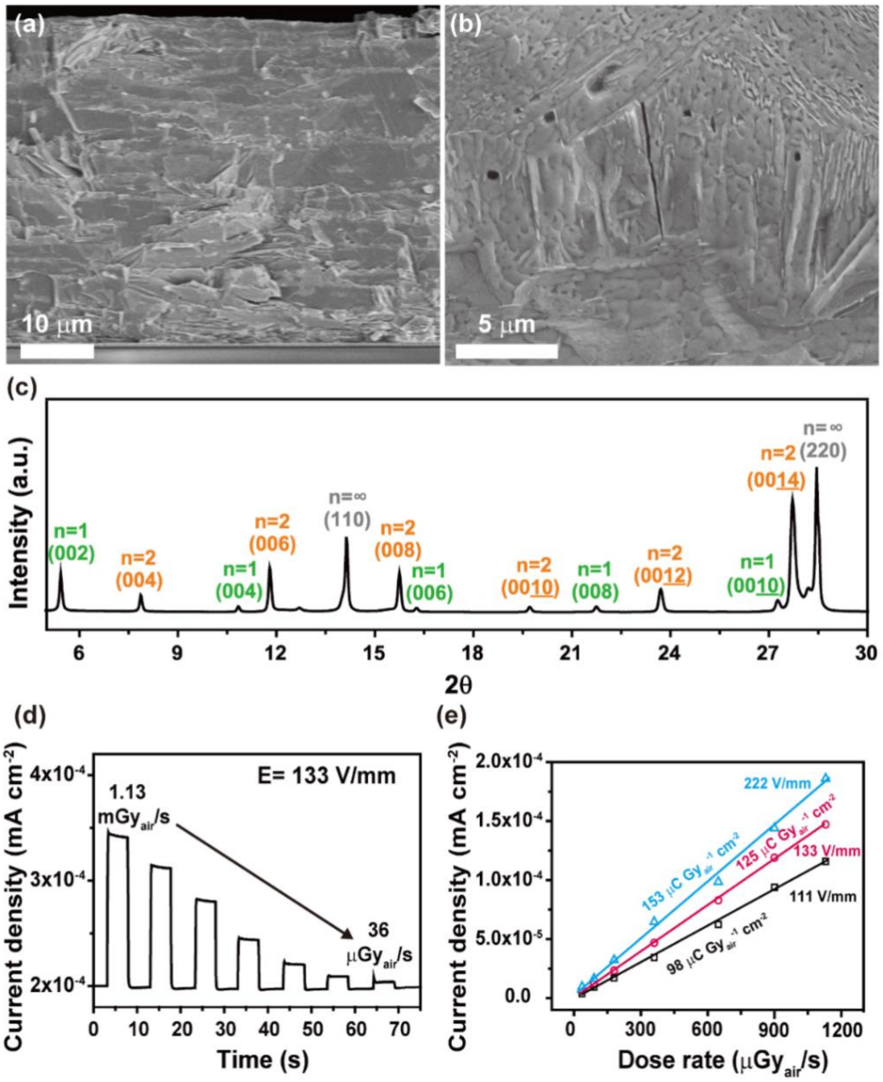

f

Figure S16. SEM images (a-b) and XRD patterns (c) of a 2D $(PEA)_2MA_3Pb_4I_{13}$ film; (d) X-ray response (d) and X-ray generated current density (e) of the detector made of a 45-μm-thick 2D $(PEA)_2MA_3Pb_4I_{13}$ film with dose rate ranging from 1.13 mGy_{air} s^{-1} to 36 μGy_{air} s^{-1}.

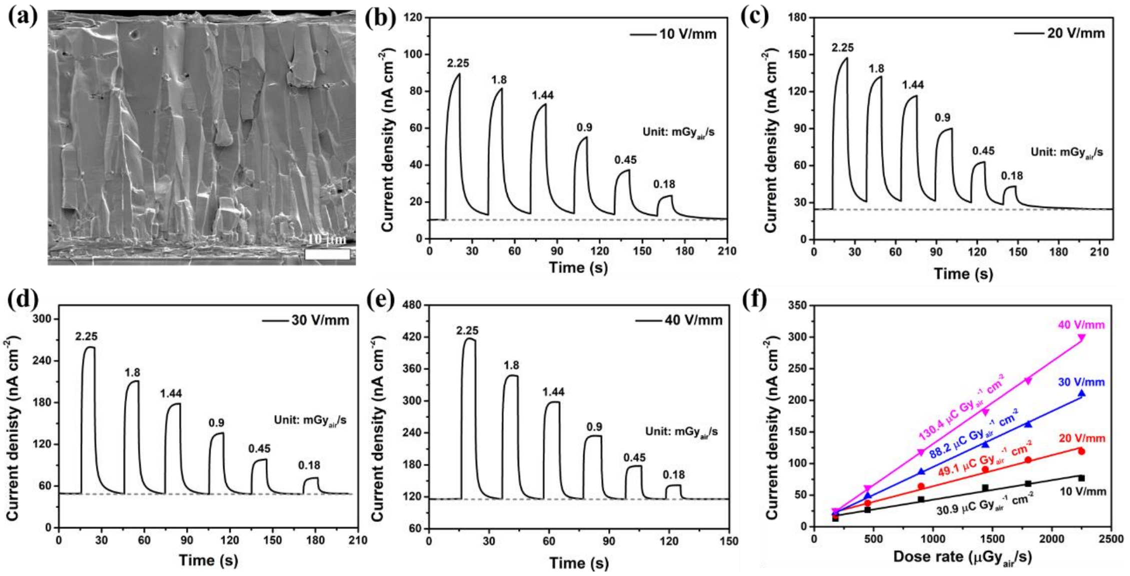

Figure S17. (a) A cross-section SEM image of the 2D/3D/2D sandwich perovskite, where the thickness of the bottom and top 2D perovskite is about 2.5 and 1.5 μm, respectively; The X-ray response characteristics of the detector made of 2D/3D/2D sandwich perovskite under different electrical felids: (b) 10 V/mm; (c) 20 V/mm; (d) 30 V/mm and (e) 40 V/mm; (f)The electrical field dependent sensitivity of the X-ray detector made of the 2D/3D/2D sandwich perovskite.

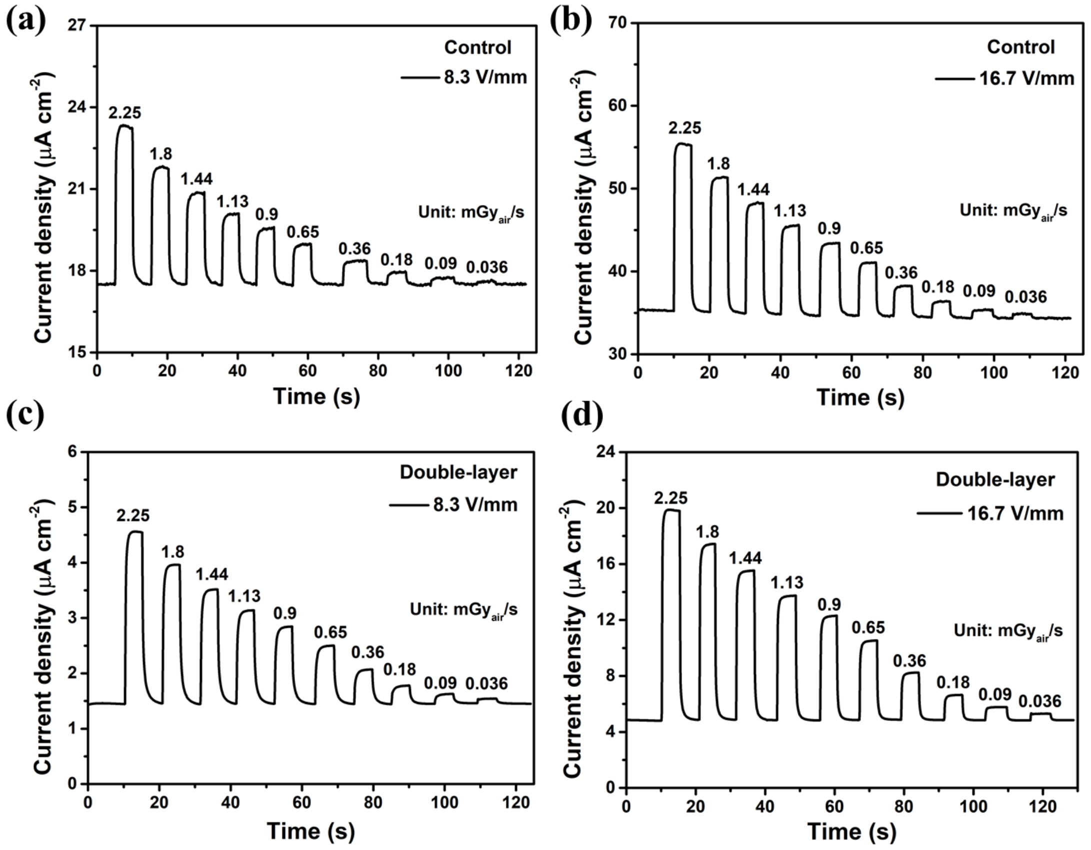

Figure S18. The X-ray response profiles of the control detector with an electrical field of 8.3 V/mm (a) and 16.7 V/mm (b); The X-ray response profiles of the 2D/3D double-layer perovskite detector with an electrical field of 8.3 V/mm (c) and 16.7 V/mm (d).

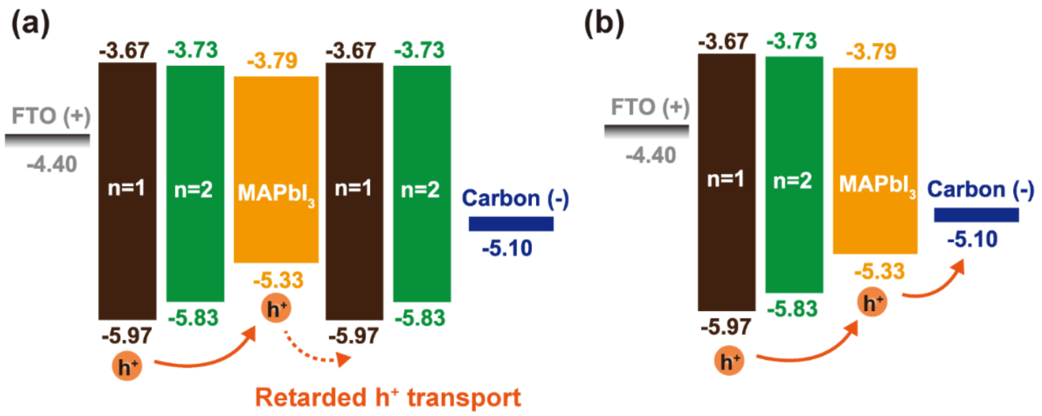

Figure S19. The energy level alignment in the detector devices made of the 2D/3D/2D sandwich perovskite (a) and the 2D/3D double-layer perovskite (b).

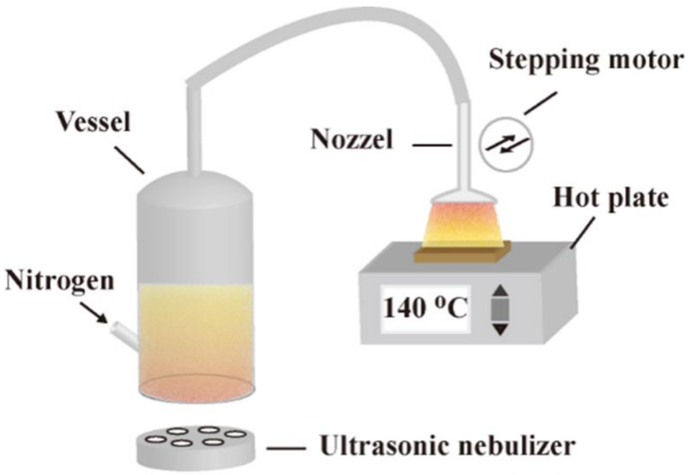

Figure S20. Schematic diagram of the ALS process.

Table S1. Summary of performance of the $MAPbI_3$ X-ray detectors reported so far.

**Device structure**	**Active layer (thickness)**	**Sensitivity [μC Gy_{air}^{-1} cm^{-2}]**	***LoD* [nGy_{air} s^{-1}]**	**Baseline drifting [nA cm^{-2} s^{-1} V^{-1}]**	**Ref**
Au/Cr/$MAPbI_3$/Cr/Au	Single-crystalline (1 mm)	968.9	Not given	Not given	Adv. Funct. Mater. 2019, 29, 1806984
Ga/$MAPbI_3$/Au	Single-crystalline (1.2 mm)	3670	19100	Not given	Angew. Chem. Int. Ed. 2019, 58, 17834
ITO/PEDOT:PSS/ $MAPbI_3$ /PCBM/ZnO/Ag	Polycrystalline (600 nm)	1.5	Not given	Not given	Nat. Photonics, 2015, 9, 444
ITO/PEDOT:PSS/ $MAPbI_3$ /PCBM/ZnO/Ag	Polycrystalline (1 mm)	2527	Not given	Obvious	Nat. Photonics, 2017, 11, 436
ITO/PI-$MAPbI_3$/$MAPbI_3$ /PI-$MAPbBr_3$/Ag	Polycrystalline (800 μm)	1.1×10^4	Not given	Not given	Nature, 2017, 550, 436, 87
Au/$MAPbI_3$/Au	Polycrystalline (800 μm)	1.22×10^5	Not given	Obvious	ACS Appl. Mater. Interfaces 2020, 12, 16592
FTO/$MAPbI_3$/C	Polycrystalline (60 μm)	2.43×10^4	3100	0.79	This work
FTO/2D/$MAPbI_3$/C	Polycrystalline (60 μm)	1.95×10^4	480	0.019	This work
